# Three Structure-Selective Endonucleases Are Essential in the Absence of BLM Helicase in *Drosophila*

**DOI:** 10.1371/journal.pgen.1002315

**Published:** 2011-10-13

**Authors:** Sabrina L. Andersen, H. Kenny Kuo, Daniel Savukoski, Michael H. Brodsky, Jeff Sekelsky

**Affiliations:** 1 Curriculum in Genetics and Molecular Biology, The University of North Carolina at Chapel Hill, Chapel Hill, North Carolina, United States of America; 2 Department of Biology, The University of North Carolina at Chapel Hill, Chapel Hill, North Carolina, United States of America; 3 Program in Gene Function and Expression, Program in Molecular Medicine, University of Massachusetts Medical School, Worcester, Massachusetts, United States of America; 4 Program in Molecular Biology and Biotechnology, The University of North Carolina at Chapel Hill, Chapel Hill, North Carolina, United States of America; National Cancer Institute, United States of America

## Abstract

DNA repair mechanisms in mitotically proliferating cells avoid generating crossovers, which can contribute to genome instability. Most models for the production of crossovers involve an intermediate with one or more four-stranded Holliday junctions (HJs), which are resolved into duplex molecules through cleavage by specialized endonucleases. *In vitro* studies have implicated three nuclear enzymes in HJ resolution: MUS81–EME1/Mms4, GEN1/Yen1, and SLX4–SLX1. The Bloom syndrome helicase, BLM, plays key roles in preventing mitotic crossover, either by blocking the formation of HJ intermediates or by removing HJs without cleavage. *Saccharomyces cerevisiae* mutants that lack Sgs1 (the BLM ortholog) and either Mus81–Mms4 or Slx4–Slx1 are inviable, but mutants that lack Sgs1 and Yen1 are viable. The current view is that Yen1 serves primarily as a backup to Mus81–Mms4. Previous studies with *Drosophila melanogaster* showed that, as in yeast, loss of both DmBLM and MUS81 or MUS312 (the ortholog of SLX4) is lethal. We have now recovered and analyzed mutations in *Drosophila Gen*. As in yeast, there is some redundancy between *Gen* and *mus81*; however, in contrast to the case in yeast, GEN plays a more predominant role in responding to DNA damage than MUS81–MMS4. Furthermore, loss of DmBLM and GEN leads to lethality early in development. We present a comparison of phenotypes occurring in double mutants that lack DmBLM and either MUS81, GEN, or MUS312, including chromosome instability and deficiencies in cell proliferation. Our studies of synthetic lethality provide insights into the multiple functions of DmBLM and how various endonucleases may function when DmBLM is absent.

## Author Summary

The maintenance of a stable genome is crucial to organismal survival. Genome stability is perpetually threatened by spontaneous DNA damage, and DNA repair proteins are required to accurately and efficiently repair DNA damage in ways that minimize genome alterations. Some repair pathways are linked to increased risk of genome changes. One example is repair associated with the production of crossovers between homologous chromosomes. The DNA helicase BLM suppresses genome changes by promoting non-crossover forms of repair; without BLM, spontaneous crossovers, deletions, and genome rearrangements increase. Using *Drosophila* as a model organism, our studies reveal the complex interactions between BLM and three structure-selective endonucleases with overlapping substrate specificities and partial functional redundancy. Loss of BLM and any one of the nucleases results in severe genome instability, reduced cell proliferation, and, ultimately, death of the animal. Our work suggests that these nucleases differentially rescue the loss of functions of BLM associated with problems that arise during DNA replication, illuminating the complexity of repair mechanisms required to maintain genome stability during replication. Further, our work advances models of replication-associated repair by suggesting specific roles for BLM and structure-selective endonucleases.

## Introduction

Crossover repair of DNA damage is associated with detrimental side effects, including loss of heterozygosity and formation of chromosome rearrangements. This genomic instability is highly deleterious, being linked to loss of cell cycle regulation and cell death; consequently, crossover (CO) formation is strongly suppressed in normal mitotic cells. One source of COs is the recombinational repair of DNA double-strand breaks (DSBs). The most widely cited model for formation of COs during DSB repair involves formation of an intermediate with two four-stranded Holliday junctions (HJs; see Figure S1) [1]. Apparent double-Holliday junction (dHJ) intermediates have been isolated as precursors of meiotic COs in *Saccharomyces cerevisiae* [2]. Similar structures are also formed during DSB repair in vegetative *S. cerevisiae* cells, though at a much lower frequency [3].

The BLM helicase has been identified as a key anti-CO factor. Mutations in the human *BLM* gene lead to Bloom syndrome, which is characterized by reduced size, fertility defects, immunodeficiency, and highly increased risk for a broad spectrum of cancers [4]. On the cellular level, *BLM* mutation increases COs between sister chromatids and homologous chromosomes, and increases the frequency of deletions and genome rearrangements [5]. This function is widely conserved, as the *S. cerevisiae* ortholog, Sgs1, also prevents COs during DSB repair [6] and the *Drosophila* ortholog, DmBLM, prevents both spontaneous and induced mitotic COs [7]. At least two models to explain the anti-CO activity of BLM have been proposed (Figure S1). First, DmBLM has been shown to promote the synthesis-dependent strand annealing (SDSA) pathway for DSB repair (Figure S1) [8]. It has been suggested that BLM's function in this pathway is to dissociate D-loops generated by strand exchange and repair synthesis [9]. Second, BLM has been proposed to catalyze convergent branch migration of the two HJs in the dHJ intermediate and facilitate subsequent decatenation by TOP3α [10]. These hypotheses are supported by *in vitro* demonstration of D-loop disruption, HJ branch migration, and, together with TOP3α and other proteins, dHJ dissolution activities [11]–[13].

In the absence of BLM, it is thought that COs may be generated through cleavage of a dHJ or similar structure by a HJ resolvase (dHJ resolution). The identity of the hypothesized HJ resolvase remains unknown, but may be one or more of the three structure-selective nuclear endonucleases that have been reported to cleave HJs *in vitro*: Mus81–Eme1/Mms4, GEN1/Yen1, and SLX4–SLX1. The first of these to be implicated in HJ resolution was Mus81-Eme1/Mms4 [14]. Mus81–Eme1 is required for most meiotic COs in *S. pombe* and a subset of meiotic COs in several other organisms [14]–[18], and appears to be involved in generating many spontaneous mitotic COs in *S. cerevisiae* [19]. However, while Mus81–Eme1/Mms4 can cut fully-ligated HJs *in vitro*, it has more robust activity on other structures, including nicked HJs, 3′ flaps, and structures that mimic replication forks [20]–[24]. Genetic studies implicate this enzyme in replication-associated repair [25]. Mammalian cells mutant for *MUS81* or *EME1* are hypersensitive to agents that generate DNA damage that blocks replication forks, such as the interstrand crosslinking agent cisplatin [26]; yeast *mus81* mutants are hypersensitive to the alkylating agent methyl methanesulfonate (MMS) and UV radiation [27]. Curiously, *Drosophila mus81* mutants do not display the same strong hypersensitivities or have defects in generating meiotic COs [28].

The second eukaryotic nuclease found to resolve HJs was initially purified from yeast (Yen1) and human cells (GEN1) by its HJ-resolvase activity. GEN1 and Yen1 both cut HJs in a symmetrical, re-ligatable manner; they also cut 5′ flap and replication fork-like structures, though not as well as they cut HJs [29]. *In vivo* functions of Yen1/GEN1 are poorly understood. *S. cerevisiae yen1* mutants are not hypersensitive to DNA damaging agents and grow normally, but *mus81 yen1* double mutants exhibit slow growth [30]. These double mutants also are more hypersensitive to MMS, HN2, camptothecin, and hydroxyurea and have fewer spontaneous mitotic COs than *mus81* single mutants [19]. *S. pombe* lacks a Yen1 ortholog, but expression of human GEN1 rescues the meiotic CO and mutagen sensitivity defects of *mus81* mutants [31]. Together, these observations suggest that Yen1/GEN1 primarily plays a backup role to Mus81–Mms4/Eme1.

The most recent enzyme reported to cut HJs *in vitro* is human BTBD12/SLX4–SLX1 [32]-[34]. Like GEN1, SLX4–SLX1 also cuts 5′ flaps and structures that mimic replication forks. Functions of this enzyme *in vivo* are also poorly understood. The *Drosophila* ortholog of SLX4 is MUS312 [35]; *mus312* mutants are hypersensitive to agents that induce DNA interstrand crosslinks (ICLs), suggesting that MUS312 is required to repair ICLs [36]. Vertebrate SLX4–SLX1 has also been implicated in ICL repair, based on hypersensitivity to crosslinking agents of cells in which either subunit is knocked down by RNAi [32]–[35]. In support of this conclusion, recent studies have identified mutations in *SLX4* in some patients with Fanconi anemia, a disorder associated with an aberrant response to crosslinking agents [37], [38]. Slx1, the catalytic subunit, appears to function solely when dimerized with Slx4, but Slx4 has other nuclease partners [39]. One of these is Rad1–Rad10, an endonuclease that functions in nucleotide excision repair [40]. These dual interactions are conserved in the *Drosophila* and vertebrate orthologs [32]–[35]. The *Drosophila* ortholog of Rad1–Rad10, MEI-9–ERCC1, is required for most meiotic COs. Interaction with MUS312 is essential for this function; it has been proposed that MUS312–MEI-9–ERCC1 generates COs by resolving HJs [41], [42], but *in vitro* analysis of this enzyme has not been published. Mouse SLX4 and the *C. elegans* ortholog HIM-18 are also involved in generating meiotic COs, though the extent to which different interacting nucleases are involved in these organisms is not yet clear [43], [44].

In fungi, simultaneous loss of both the BLM helicase ortholog, Sgs1, and either Mus81–Eme1/Mms4 or Slx4–Slx1 is lethal [45], [46], [47]. Studies of these synthetic lethal phenotypes have provided additional insights into functions of Sgs1/BLM and these putative HJ resolvases. Likewise, previous studies revealed that mutations in *Drosophila mus309*, the gene that encodes DmBLM, are synthetically lethal with mutations in *mus81* or *mus312* [28], [35], [48]. Genetic interactions have also been observed in Bloom syndrome cells in which these nucleases were knocked down singly or in combinations, ranging from modest decreases in sister chromatid exchange to chromosome fragmentation and decreased cell viability [49].

We have now obtained mutations in *Drosophila slx1* and *Gen*. We find that *slx1 mus309* mutants are inviable, with phenotypes similar to those of *mus312 mus309* mutants. As in yeast, GEN and MUS81 have some overlapping or compensatory functions; however, *Gen* mutants have more severe hypersensitivities than *mus81* mutants, suggesting that GEN plays a more critical role in DNA repair in *Drosophila*. In support of this conclusion, *Gen mus309* mutants are inviable, and die much earlier in development than either *mus81*; *mus309* or *mu312 mus309* double mutants. Therefore, three putative HJ resolvases – MUS81–MMS4, GEN, and MUS312–SLX1 are essential in the absence of DmBLM. Each of the double mutants has defects in cell proliferation and features of chromosome instability, though the severities vary from genotype to genotype. The effects of blocking recombination by mutating the gene encoding the ortholog of the strand exchange protein Rad51 (SPN-A in *Drosophila*) also vary, from nearly complete suppression of defects to selective suppression of a subset of phenotypes. We also analyzed the effects of a *mus309* mutation that abolishes a subset of DmBLM functions. Together, our results suggest models for functions of DmBLM in responding to spontaneous replication fork problems, and how MUS81–MMS4, GEN, and MUS312–SLX1 might function in alternative, DmBLM-independent pathways.

## Results

### *mus312 mus309* Synthetic Lethality Is Due to Loss of MUS312-SLX1

We previously reported synthetic lethalities between mutations in *mus309*, which encodes the *Drosophila* ortholog of the BLM helicase, and mutations in *mus81* or *mus312* [28], [35], which encode subunits of putative HJ resolvases [14], [32], [33], [34]. MUS81 is the catalytic subunit of a heterodimeric endonuclease (MUS81–MMS4). MUS312 is a non-catalytic subunit that interacts with at least two nucleases, SLX1 and MEI-9–ERCC1 [35]. It seemed likely that the *mus312 mus309* lethality is due to loss of the MUS312–SLX1 nuclease, since *S. cerevisiae slx1 sgs1* double mutants are inviable and have phenotypes similar to those of *slx4 sgs1* double mutants [47]. To determine the contributions of SLX1 and MEI-9–ERCC1 to *mus312 mus309* lethality, we generated double mutants between each of them and *mus309*. We found that *mei-9*; *mus309* double mutants are viable, consistent with the previous finding that MEI-9–ERCC1 interacts with MUS312 in meiotic recombination, but not in somatic DNA repair [42].

Mutations in *Drosophila slx1* (*CG18271*) have not been reported previously. A complication in generating a mutation in this gene is that the first (non-coding) exon overlaps the first exon of *MED31*, which is thought to be an essential gene [50]. We therefore generated a synthetic deletion by combining a 30-kb chromosomal deficiency with a transgene that spans the region but on which we disrupted *slx1* (Materials and Methods, Figure S2). As an alternative approach, we ordered Targeted Induction of Local Lesions in Genes (TILLING) from the Seattle Drosophila TILLING Project [51]. This effort identified 24 missense mutations in *slx1*, the most promising being one that changes a conserved phenylalanine residue to isoleucine (F93I; Figure S2). Both *slx1^F93I^* and the synthetic deletion are lethal when combined with *mus309* mutations. Double mutants die early in the pupal stage, larvae lack imaginal discs, and larval neuroblasts are frequently polyploid. These phenotypes are similar to those of *mus312 mus309* double mutants [35, see below]. We conclude that the inviability of *mus312 mus309* double mutants is indeed due to simultaneous loss of DmBLM and MUS312–SLX1. In experiments described below, we used *mus312 mus309* mutants to further characterize defects caused by loss of DmBLM and MUS312–SLX1.

### MUS81–MMS4 and GEN Have Overlapping Functions

Orthologs of MUS81–MMS4 and MUS312–SLX1 have been implicated in HJ resolution. We therefore wanted to determine whether GEN, which is orthologous to the HJ resolvases Yen1 and GEN1, is also essential when DmBLM is absent. *Drosophila* GEN was initially identified as a novel RAD2/XPG family nuclease [52]. GEN was found to cut flaps and the lagging strand of replication fork-like structures, as well as to have a weak exonuclease activity on nicked substrates [53]. However, no stocks carrying *Gen* mutations have been reported. To identify such mutations, we screened through a collection of mutagen-sensitive (*mus*) lines for which the causative mutations had not been mapped [54]. We discovered that the two available *mus324* stocks both have mutations in *Gen*. Although the two alleles, *mus324^Z4325^* and *mus324^Z5997^*, were assumed to be independent, they have identical mutations (deletion of ATATAC and insertion of a single G, creating a frameshift at codons 374-5, which is within the conserved nuclease domain), suggesting that they are two isolates of the same mutational event. *mus324* mutants are hypersensitive to the crosslinking agent HN2 and to the alkylating agent MMS [54]; we found that these hypersensitivities are uncovered by *Df(3L)Exel6103*, a deletion that removes *Gen* and 16 other genes (data not shown). We conclude that *mus324* is *Gen* and hereafter refer to these alleles as *Gen^Z4325^* and *Gen^Z5997^*.

**Figure 1:**
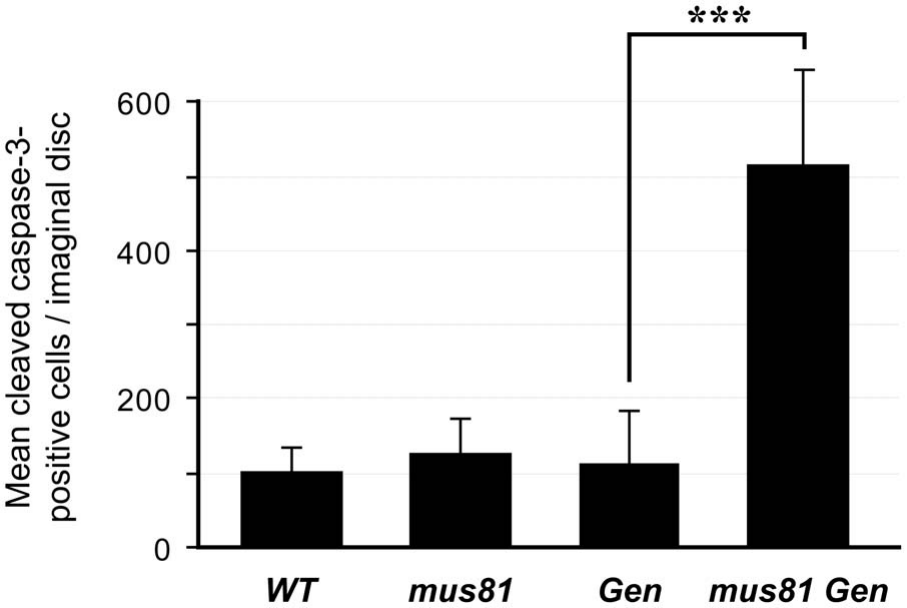
Apoptosis in larval imaginal discs. Apoptosis levels are expressed as the average number of cells per imaginal wing disc that stain with antibody to cleaved caspase-3. n = number of discs scored. *mus81* and *Gen* mutant larvae had apoptosis levels indistinguishable from the wild-type control (*y w*), but *mus81 Gen* double mutants had significantly increased apoptosis compared to either wild-type or either of the single mutants. *** p<0.0001 (Fisher's exact test).

In contrast to the situation in *S. cerevisiae*, *Drosophila Gen* mutants are more hypersensitive to MMS and HN2 than *mus81* mutants [28,54, S. Bellendir and JS, unpublished data]. As in *S. cerevisiae*, however, there appears to be overlapping functions for these two nucleases. In the absence of exogenous DNA damage, *mus81*; *Gen* double mutants have wild-type survival rates relative to heterozygous siblings, but the eyes of double mutants are reduced in size and exhibit mild roughening (data not shown). This phenotype often results from cell cycle defects and/or increased apoptosis disrupting the highly ordered ommatidia. We quantified apoptosis in larval imaginal discs, which consist of proliferative diploid cells that give rise to adult epidermal structures such as eyes, wings, and legs. Imaginal wing discs from *mus81* and *Gen* single mutants have the same levels of apoptosis as wing discs from wild-type larvae, but double mutants have significantly increased levels (Figure 1). The high level of apoptosis in the double mutant imaginal discs suggests that MUS81 and GEN have shared functions that contribute to cell survival or proliferation even in the absence of exogenously-induced DNA damage.

### Loss of DmBLM and GEN Is Lethal

**Figure 2:**
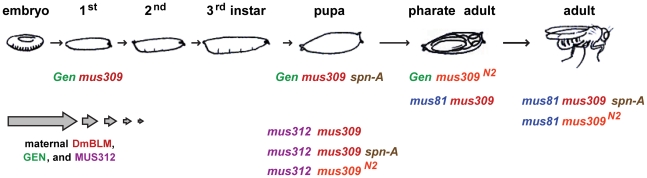
Lethal stages of various mutants. The *Drosophila* life cycle is illustrated, with the lethal stages of different genotypes indicated. The arrows at the bottom left are intended to signify diminishing contribution of maternally-deposited protein. In our crosses, there is no maternal MUS81, but there is half the normal amount of maternal DmBLM, MUS312, and GEN; the developmental stage to which this maternal protein perdures is unknown, and may be different in different tissues.

Knowing that two putative HJ resolvases (MUS81–MMS4 and MUS312–SLX1) are required when DmBLM is absent, we wanted to determine whether GEN is also essential when DmBLM is absent. We generated *Gen mus309* double mutants and found that they die early in larval development, reaching only the first instar stage (Table 1; Figure 2). This is earlier in development than *mus81*; *mus309* double mutants, which die as pharate adults (adult structures such as wings and eyes are visible within the pupal case, but no adults eclose), and also earlier than *mus312 mus309* double mutants, which die at an early pupal stage. As a consequence of the genetic crosses we used (see Materials and Methods), the *mus81*; *mus309* double mutants we analyzed have no maternal contribution of MUS81, but both *Gen mus309* and *mus312 mus309* double mutant larvae potentially have maternally-deposited wild-type GEN and MUS312 (there is maternal DmBLM in all three cases). The weaker lethal phenotype of *mus81*; *mus309* mutants is therefore consistent with MUS81 contributing less to DmBLM-independent pathways than either GEN or MUS312.

**Table 1:** Comparison of mutant phenotypes.

Genotype	lethal stage	brain size	imaginal discs	salivary imaginal ring cell nuclei	neuroblast ploidy
wild type	viable	+	+	+	+
*mus309*	viable	+	+	+	+
*mus81*	viable	+	+	+	+
*mus81; mus309*	pharate	+	+	+	+
*mus81; mus309 spn-A*	viable	+	+	+	+
*mus81; mus309^N2^*	viable	+	+	+	+
*mus312*	viable	+	+	+	+
*mus312 mus309*	pupal	**↓**	–	↓ number, ↑ size	↑
*mus312 mus309 spn-A*	pupal	**↓**	–*	+	+
*mus312 mus309^N2^*	pupal	**↓**	–*	+	+
*Gen*	viable	+	+	+	+
*Gen mus309*	1^st^ instar	ND	ND	ND	ND
*Gen mus309 spn-A*	pupal	**↓**	**–** *	↓ number, ↑ size	+
*Gen mus309^N2^*	pharate	+	**+**	+	+

+  =  wild-type; **–**  =  absent; **↓**  =  decreased; ↑  =  increased; ND  =  not determined (due to early larval lethality);

* =  very small discs occasionally seen.

### Proliferation Defects in Double Mutants

**Figure 3:**
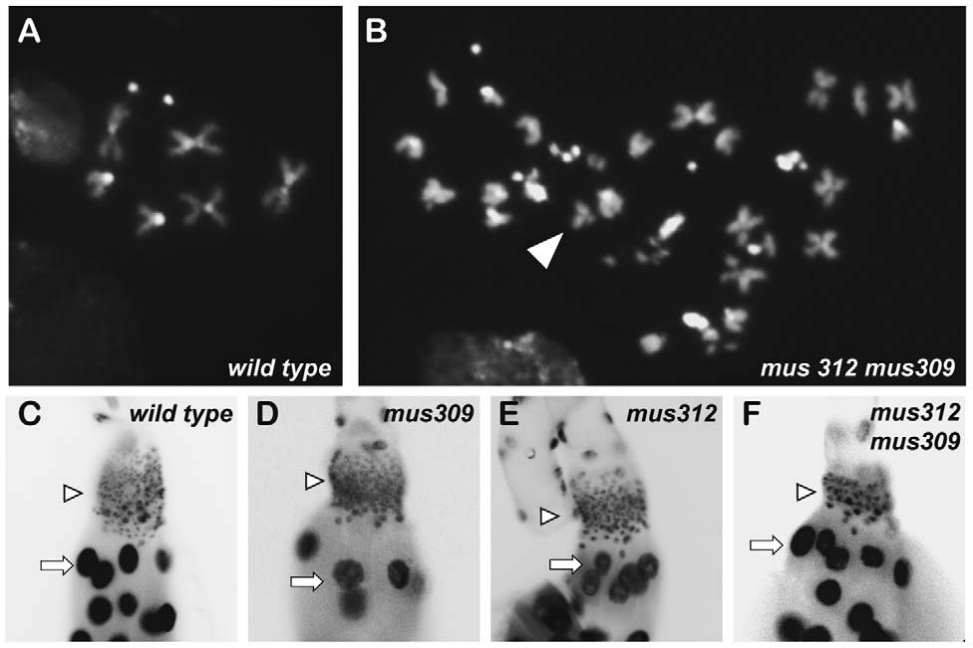
Nuclear defects in mutants. (A-B) DAPI-stained metaphase neuroblast nuclei. Normal wild-type nuclei (A) contain one pair of sex chromosomes, two pairs of large autosomes, and one pair of small autosomes. Most nuclei of single mutants for *mus81*, *mus309*, *Gen*, or *mus312* are normal (see Figure 4). (B) An example of a cell from a *mus312 mus309* mutant, illustrated polyploidy and broken chromosomes (arrowhead). (C-F) DAPI-stained larval salivary gland nuclei. Arrows point to polyploid nuclei; arrowheads point to diploid imaginal nuclei. Compared to wild-type (C) and *mus309* (D) and *mus312* (E) single mutants, diploid imaginal ring cell nuclei are reduced in number and enlarged in size in *mus312 mus309* double mutants (F) and in *Gen mus309 spn-A* (not shown). Endocycling polytene cells are similar in all genotypes shown.

To gain more insights into the causes of the three synthetic lethalities, we examined several highly proliferative larval tissues. Although most larval growth is due to enlargement of cells undergoing endocycles without mitosis, there is extensive cell proliferation in several tissues, including the neuroblasts of brain, the imaginal discs, and cells in the imaginal ring of the salivary gland. These tissues all appear to be normal in *mus81*; *mus309* double mutants. In contrast, *mus312 mus309* mutants have small brains, lack imaginal discs, and have a reduced number of salivary imaginal cells (Figure 3). Nuclei of the remaining salivary imaginal cells appear larger than in wild-type larvae, suggesting increased DNA content. These phenotypes indicate that MUS312–SLX1 has a more critical role in proliferation in the early larva than MUS81–MMS4 (Table 1). *Gen mus309* mutants die too early to examine these tissues; it is likely that this early lethality is due to an even more severe defect in cell proliferation.

### Chromosome Defects in Double Mutants

**Figure 4:**
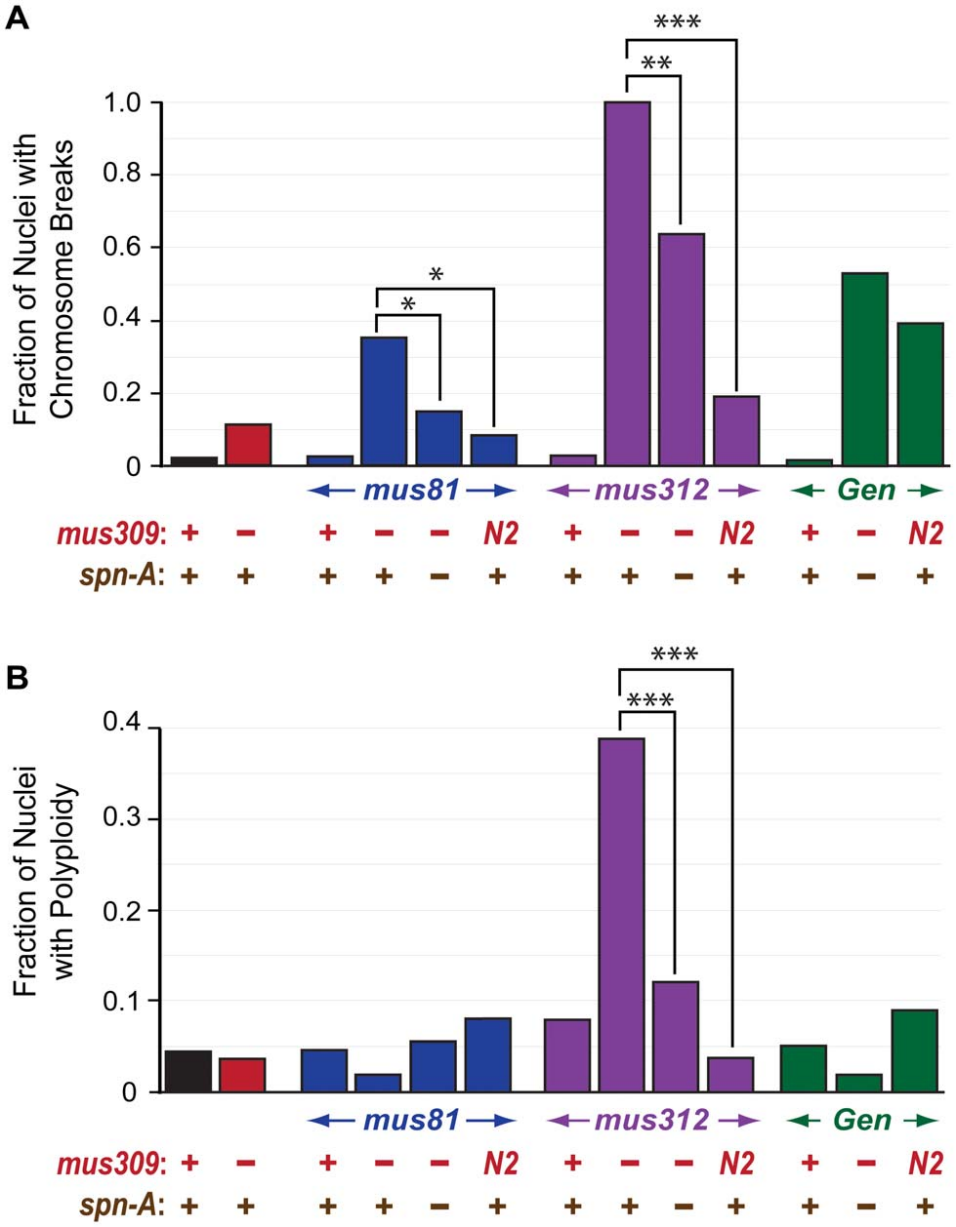
Chromosome breaks and polyploidy in mutants. A. Fraction of chromosomes in metaphase neuroblast nuclei with breaks. B. Fraction of metaphase neuroblast nuclei with polyploidy in different genotypes. * p<0.05, ** p<0.01, *** p<0.0001 (Fisher's exact test). Number of nuclei scored (left to right): 46, 27, 44, 54, 24, 54, 38, 18, 33, 26, 59, 53, 43.

We hypothesized that the proliferation defects described above stemmed, at least in part, from unrepaired DNA damage and/or unresolved DNA repair intermediates. To determine whether there were gross chromosomal changes in the double mutants, we arrested larval neuroblasts with colchicine and examined mitotic nuclei for chromosome structural damage and aneuploidy. The frequencies of chromosome aberrations and aneuploidy in mitotic neuroblasts were indistinguishable between wild-type and single mutants for *mus81*, *mus312*, *mus309*, or *Gen* (Figure 3, Figure 4). In *mus81*; *mus309* double mutants, there was an increased frequency of broken chromosomes (Figure 4A). The *mus312 mus309* double mutants showed extreme genome instability: No mitotic nuclei with completely intact chromosomes were detected, and about a third showed polyploidy (Figure 3B, Figure 4). Precise chromosome counts could not be made because of the highly fragmented nature of the chromosomes, but some nuclei appeared to be more than 4N (tetraploid). The early larval death of *Gen mus309* mutants precluded us from examining neuroblast chromosomes in this genotype.

### Preventing Strand Exchange Suppresses Specific Phenotypes in Double Mutants

In *S. cerevisiae*, synthetic lethality between *sgs1* and *mus81* is suppressed by *rad51* mutations, but lethality between *sgs1* and *slx4* is not [45], [55]. The *Drosophila* ortholog of the strand exchange protein RAD51 is encoded by *spn-A* [56]. As in yeast, mutation of *spn-A* suppresses the lethality of *mus81*; *mus309* mutants [28]. A simple interpretation is that strand exchange mediated by SPN-A leads to a toxic intermediate that must be processed by either DmBLM or MUS81; however, a more thorough analysis of multiple genotypes suggested a more complex model [28]. Suppression of lethality of *mus81*; *mus309* by *spn-A* is not complete, since the triple mutant still has increased apoptosis relative to wild-type larvae and only 70% of the triple mutants survive to adulthood [28]. We found that loss of *spn-A* also leads to a significant decrease in the number of chromosome breaks in neuroblast cells of *mus81*; *mus309* mutants (Figure 4A). As with viability and apoptosis, suppression is not complete, which suggests that either a subset of the damage that DmBLM and MUS81 are required to process is not generated through a SPN-A-dependent process, or that pathways that operate when SPN-A is unavailable are not sufficient to repair all the spontaneous damage that would normally be processed through strand exchange-mediated pathways.

Mutation of *spn-A* has a similarly pronounced effect on the phenotype of *Gen mus309* mutants. Rather than dying at the first larval instar, the *Gen mus309 spn-A* mutants survive to the pupal stage (Table 1). Third instar triple mutant larvae have small brains and lack imaginal discs, though very small rudimentary discs are occasionally visible. Salivary imaginal cells are reduced in number and have enlarged nuclei (Table 1). Neuroblasts from *Gen mus309 spn-A* larvae have numerous chromosome breaks (Table 1, Figure 4A). Thus, preventing strand exchange ameliorates the phenotypes caused by loss of GEN and DmBLM, but the triple mutants still have cell proliferation defects.

In contrast to the profound suppression of defects that arise when DmBLM and either MUS81 or GEN are absent, preventing strand invasion suppressed only some defects caused by loss of both MUS312 and DmBLM. Like *mus312 mus309* double mutants, *mus312 mus309 spn-A* triple mutants lack imaginal discs, have small brains, and die at the pupal stage (Table 1). However, mutation of *spn-A* does suppresses the chromosome breakage or polyploidy phenotypes (Figure 4), as well as the defect in salivary gland imaginal cells (data not shown). The differences in the effect of *spn-A* mutations on the various phenotypes suggests that there are multiple circumstances in which either DmBLM or MUS312–SLX1 are essential, but that only a subset of these result from strand exchange.

### A *mus309* Separation-of-Function Allele Has Less Severe Phenotypes in Double Mutants

Trowbridge *et al* [28] reported that *mus81* mutations are viable with the separation-of-function allele *mus309^N2^*. This mutation is an intragenic deletion predicted to remove the first 566 residues of DmBLM but leave the helicase domain intact [7], [28]. Previous studies showed that *mus309^N2^* are similar to null mutants in their inability to repair DNA double-strand gaps by SDSA, their hypersensitivity to ionizing radiation, and their elevated levels of spontaneous mitotic COs [7]. However, maternal-effect embryonic lethality, which is associated with extensive anaphase bridging in early-stage embryos, is substantially reduced in *mus309^N2^* mutants compared to null mutants, though not to wild-type levels [7]. We hypothesized that DmBLM is required during the extremely rapid early embryonic S phases, particularly in the decatenation of converging replication forks, and that DmBLM^N2^ is capable of carrying out this process, though not with wild-type efficiency [7]. This led to the suggestion that an important function revealed by *mus81*; *mus309* lethality is in processing blocked or regressed replication forks, either by DmBLM-catalyzed migration or by MUS81-dependent cleavage. In this model, *mus81*; *mus309^N2^* mutants are viable because DmBLM^N2^ retains the ability to migrate these forks. Thus, the alleviation of maternal-effect lethality in *mus309^N2^* females and the viability of *mus81*; *mus309^N2^* double mutants suggests that DmBLM^N2^ retains some replication-fork processing functions. In contrast, the null-equivalent defect in SDSA in *mus309^N2^* mutants suggests that the ability to disrupt D-loops during SDSA is destroyed in DmBLM^N2^, while the null-equivalent elevation in mitotic COs suggests that DmBLM^N2^ is also unable to catalyze dHJ dissolution.

To determine the extent to which the activities retained by DmBLM^N2^ can compensate for the loss of GEN or MUS312–SLX1, we made *Gen mus309^N2^* and *mus312 mus309^N2^* double mutants. G*en mus309^N2^* mutants are inviable. However, rather than dying as first instar larvae, *Gen mus309^N2^* mutants die later, as pharate adults. These double mutants have apparently normal imaginal disc size, brain size, and number/size of salivary gland imaginal cells (Table 1), but their neuroblasts frequently exhibit chromosome breaks (Figure 4A). The striking differences between *Gen mus309* and *Gen mus309^N2^* mutants in their cell proliferation phenotypes and stages of lethality suggest that GEN has an important role in processing replication-associated structures when DmBLM is not available, consistent with the known biochemical activities of GEN [29].

*mus312 mus309^N2^* mutants are also inviable, and are similar to double mutants between *mus312* and null alleles of *mus309* in that larvae lack imaginal discs and lethality occurs in the pupal stage (Table 1). However, several mutant phenotypes are less severe in *mus312 mus309^N2^* double mutants. Small, severely underdeveloped imaginal discs are sometimes detected in third-instar larvae, and in metaphase neuroblasts there are fewer damaged chromosomes and polyploidy is not seen (Figure 4). These observations suggest that defects in replication contribute to the chromosome breaks, polyploidy, and, perhaps stemming from these aberrations, proliferation defects that are seen in *mus312 mus309* double mutants.

*Gen mus309^N2^* mutants have fewer chromosome breaks than *mus312 mus309^N2^* mutants (P = 0.3; P = 0.35 for the differences in frequency of polyploidy), but the latter die earlier. Therefore, chromosome breaks in neuroblasts are not the sole cause of lethality. The early pupal lethality of *mus312 mus309^N2^* mutants is most likely due to the absence of imaginal discs; the reasons for the loss of this tissue are unknown, but are likely due to poor cell proliferation, elevated apoptosis, or both.

## Discussion

Our studies of synthetic lethality show that at least three different structure-selective endonuclease are crucial for processing structures that persist or arise when DmBLM is absent. In the absence of induced damage, there are no obvious defects in proliferation in *mus81*, *Gen*, or *mus312* single mutants, but apoptosis is significantly elevated in *mus309* single mutants [28]. These observations suggests that DmBLM has several important functions that operate in the absence of damage induction by exogenous agents, and that the synthetic lethalities we have described are due to loss of secondary, DmBLM-independent pathways. Although our data do not directly implicate specific function, previous studies indicate that BLM functions primarily during S phase, most likely in repair or maintenance of blocked or damaged forks [57]. Based on these considerations, and drawing from previously published models for replication fork repair [61], we suggest functions for DmBLM, MUS81–MMS4, GEN, and MUS312–SLX1 in replication fork management.

### Overlapping Functions of GEN and MUS81

In *S. cerevisiae*, *mus81 yen1* double mutants have a slow growth phenotype [58], and we found that *Drosophila mus81*; *Gen* double mutants have elevated levels of apoptosis. Thus, in both budding yeast and flies, simultaneous loss of MUS81–MMS4 and Yen1/GEN leads to spontaneous defects in cell proliferation. Although this suggests some functional overlap, the relative contributions of the two enzymes appears to be reversed in these organisms. In yeast, *mus81* single mutants are hypersensitive to a number of DNA damaging agents, but *yen1* mutants are not [19], [27], [30], [58], whereas in flies, *Gen* mutants have severe hypersensitivities and *mus81* mutants have only weak hypersensitivities [28, 54, S. Bellendir and JS, unpublished data]. It has been proposed that Mus81–Mms4 cuts nicked HJs, but if left uncut (as in *mus81* mutants), these are ligated into intact HJs that are cleaved by Yen1 [30], [31], [49].

**Figure 5:**
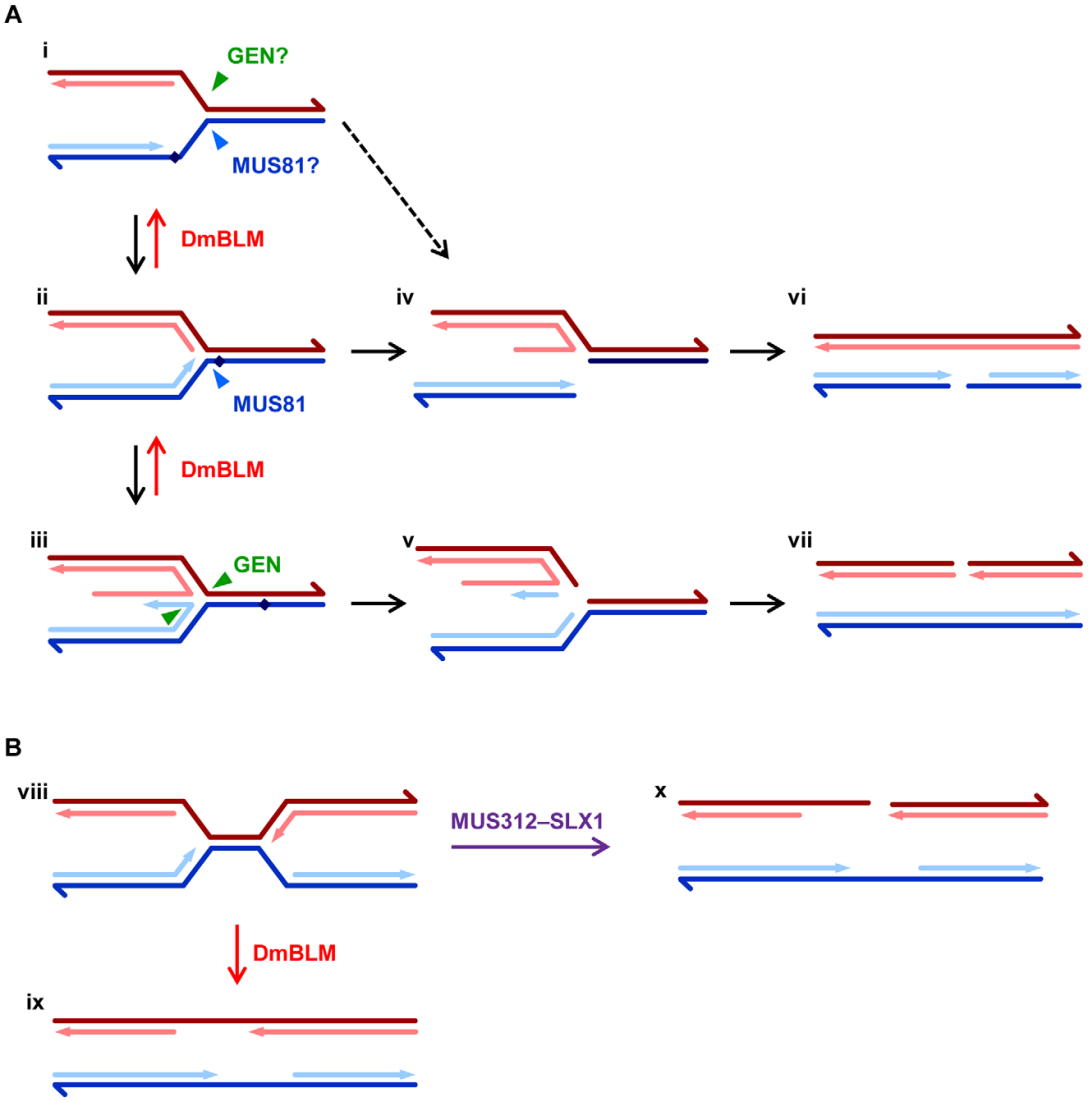
Models for roles of DmBLM and endonucleases in replication fork repair. A. The first structure (i) represents a replication fork with a block (diamond) on the leading strand. Arrowheads on dark lines indicate the 3′ ends of the template strands; arrows on light lines indicate 3′ ends of the nascent leading (blue) and lagging (red) strands. It is possible that blocked forks can be cleaved on the lagging strand template by GEN or on the leading strand template by MUS81–MMS4. More typically, however, the fork is regressed (ii), possibly with template switching (iii). After removal of the block, DmBLM catalyzes reversal of the regressed structure to re-establish the replication fork. In the absence of DmBLM, regressed forks without or with template switching can be cut by MUS81–MMS4 or GEN, respectively (iv and v). Blocked forks can also spontaneously break (dotted line), especially if not protected by SPN-A. Collapsed forks resemble one-ended DSBs, but replication from a fork to the right converts these into DSBs (vi and vii), which are repaired by standard DSB repair pathways (see Figure S1). B. Converging replication forks (viii) sometimes experience problems that are solved through a DmBLM-dependent migration/decatenation process (ix). In the absence of DmBLM, MUS312–SLX1 cuts a fork, generating a DSB (x). It is also possible that both forks are cut, leading to DSBs on both chromatids (not shown). These could both be repaired using the homologous chromosome, except in the case of the male *X* or *Y* chromosome.

The *in vitro* biochemical activities of GEN and MUS81 and the drug sensitivities of single mutants suggest that these enzymes function in replication fork repair. GEN and MUS81–MMS4 cut different sides of replication fork-like substrates *in vitro*. Functional redundancy could be explained by the ability of either to cut blocked forks (Figure 5A, i); however, in both yeast and *Drosophila* one enzyme plays a larger role in surviving exogenous DNA damage, suggesting that these enzymes act on structures other than simple stalled forks. An obvious candidate is a regressed fork. Based on *in vitro* activities, MUS81–MMS4 would be expected to have a preference for forks that are regressed but have not undergone template switching (Figure 5A, ii), whereas GEN would be expected to cut regressed forks in which the leading strand has undergone template switching (Figure 5A, iii). The different biases in enzyme preference might be explained by differing degrees of forks regression in *Saccharomyces* versus *Drosophila*.

This model assumes that *Drosophila* GEN, like Yen1 and human GEN1, is an HJ resolvase. The question of whether GEN is a resolvase has important implications for understanding the partial redundancy between *Drosophila* GEN and MUS81–MMS4. The rescue of *S. pombe mus81* mutant phenotypes by human GEN1 and studies of knockdown of these enzymes in human cells have been interpreted with respect to the HJ-cutting activities [30], [49]; however, a previous study of *Drosophila* GEN did not detect resolvase activity [53]. That study employed full-length GEN; it is possible that, like human GEN, the unstructured C-terminus must be removed to allow HJ cleavage *in vitro* [29]. Regardless of whether GEN cuts HJs, it remains possible that the genetic overlap between MUS81 and GEN is due at least in part to cleavage of other substrates that might arise during recombination or replication fork repair.

### Roles of GEN and MUS81–MMS4 as Alternatives to DmBLM

Given that GEN and MUS81–MMS4 have some overlapping function(s) and *yen1 sgs1* double mutants are viable in *S. cerevisiae* [30], we were surprised to discover that *Gen mus309* double mutants are inviable. In fact, of the three synthetic lethalities we characterized, *Gen mus309* double mutants have the most severe developmental phenotype. This suggests that the structures upon which GEN can act are more frequently produced and/or more deleterious when left unprocessed by DmBLM. Conversely, *mus81*; *mus309* mutants die the latest in development and have the least severe defects in proliferation and chromosome stability, suggesting that structures upon which MUS81–MMS4 acts are less frequently produced and/or less deleterious when left unprocessed, or that there are additional repair options available.

Insights into the nature of the structures upon which either DmBLM or one of these endonucleases can act comes from the finding that double mutants with *mus309^N2^* have much milder defects than double mutants with null alleles of *mus309* (Table 1, Figure 4). *mus309^N2^* is thought to abolish the DSB repair and dHJ dissolution functions while leaving some replication fork function(s) largely intact [7]. This suggests that synthetic lethality between *Gen* and *mus309* and between *mus81* and *mus309* are not due to inability to dissolve dHJs or disrupt D-loops, but to inability to process replication fork structures. Additional clues come from the observation that preventing strand exchange partially rescues *Gen mus309* and *mus81*; *mus309*. In both cases, every phenotype we studied is affected, though rescue is incomplete for each. Incomplete rescue may be because the repair methods that do not rely on strand exchange are themselves problematic, or because some repair intermediates that require either DmBLM or one of these endonucleases are generated by stand exchange and some are not.

A model that is consistent with our findings is illustrated in Figure 5A. It is believed that when a replication fork encounters a block to leading strand synthesis, the fork is regressed so that it is stabilized and so the blockage is accessible for removal (Figure 5A, ii). In some cases, the nascent leading strand may anneal to the nascent lagging strand (Figure 5A, iii). This template switch allows the leading strand to be extended, so that after reversal of the regression the block is bypassed. We hypothesize that DmBLM is required for reversal of regression. Forks that are regressed to various degrees might be cleaved by MUS81–MMS4 (Figure 5A, iv) or by GEN (Figure 5A, v). Regressed forks that are not reversed or cut are toxic and trigger apoptosis. In the absence of both DmBLM and MUS81–MMS4, template switching is still an option, but in the absence of both DmBLM and GEN, there are no further options; hence, *Gen mus309* mutants have a more severe defect than *mus81*; *mus309* mutants. DmBLM^N2^ is capable of reversing regressed forks, and although its activity is less than that of full-length DmBLM, it is sufficient to allow survival of most *mus81*; *mus309^N2^* individuals to adulthood, and survival of *Gen mus309^N2^* to the pharate adult stage.

Some studies suggest that Rad51 is required to protect blocked forks and perhaps to carry allow regression [59], [60], [61]. If this is true in *Drosophila*, then mutation of *spn-A* may suppress defects in double mutants by preventing fork regression, thereby blocking buildup of toxic structures. Blocked forks that are not protected by SPN-A may spontaneously break, giving rise to structures that are similar to those generated by MUS81–MMS4 or GEN cleavage (Figure 5A, dotted line). Several models have been proposed to explain how these broken forks are repaired to allow replication restart [62]. These typically involve strand invasion from the broken end into the intact sister chromatid. In *Drosophila*, however, we propose that continued replication from adjacent forks or from *de novo* firing of nearby origins converts the one-ended DSB into a two-ended DSB (Figure 5A, vi, vii). This proposal is consistent with the finding that substantial DNA synthesis persists after induction of S-phase damage in *Drosophila* [63]. Repair of the two-ended DSB would typically occur through DmBLM-dependent SDSA (see Figure S1). However, if DmBLM is not available to promote SDSA, repair occurs through a pathway that may generate a CO. As a consequence of pairing of homologous chromosomes in proliferating cells in *Drosophila*, repair will often involve interaction between homologs; this can contribute to the high elevation in mitotic COs in *mus309* mutants [7]. In the most popular models, COs arise through resolution of HJ intermediates. It is possible that GEN plays a role in this process and that this also contributes to the early lethality of *Gen mus309* double mutants.

### Roles of MUS312–SLX1 as an Alternative to DmBLM

The *mus312 mus309* synthetic lethality we describe is unique in that it is not alleviated by blocking strand exchange. This has also been reported for *S. cerevisiae sgs1 slx4* lethality [47]. Fricke *et al.* [39] proposed that an important overlapping function between Sgs1 and Slx4–Slx1 is in rDNA replication: Sgs1–Top3 decatenates forks that stall during rDNA replication, but in the absence of Sgs1 these structures are cut by Slx4–Slx1. A similar model has been suggested in *S. pombe* [64]. McVey *et al.* [7] hypothesized that DmBLM–TOP3α is required to decatenate converging replication forks during the extremely rapid S phases of early embryonic development. At this stage of development, DNA repair processes seem to be disabled [65], so maternally-deposited DmBLM is essential for early embryonic replication. We hypothesize that DmBLM is still involved in decatenation of problematic fork convergences at later developmental stages, but that DmBLM is no longer essential because a secondary pathway is available: cleavage by MUS312–SLX1 (Figure 5B). Since converging forks are not generated by strand exchange, prevention of strand exchange (through mutation of *spn-A*) does not rescue lethality. Likewise, *mus312 mus309^N2^* mutants remain inviable because DmBLM^N2^ is predicted to be unable to interact with TOP3α [7], an interaction that is expected to be essential for decatenation of converging forks.

Interestingly, the chromosome breakage and aneuploidy phenotypes are milder in *mus312 mus309^N2^* and in *mus312 mus309 spn-A* than in *mus312 mus309* null alleles (Table 1, Figure 4). This suggests that there are additional structures, generated by strand exchange but on which DmBLM^N2^ cannot act, that can be cleaved by MUS312–SLX1. One potential additional function for SLX4–SLX1 is in repairing DNA ICLs [32], [33], [34], [35], [42]. Given the HJ resolvase activity of human SLX4–SLX1, it seems plausible that MUS312–SLX1 cuts a single HJ intermediate or replication fork-like structures formed during ICL repair. It is unclear what defect leads to polyploidy. It has been suggested that prolonged blocks to the completion of DNA replication might prevent cytokinesis, leading to tetraploidy [66]. Consistent with this hypothesis, defects in S-phase-coupled processing of histone mRNAs leads to tetraploidy in *Drosophila* neuroblasts [67].

### Concluding Remarks

We've established that MUS81–MMS4, GEN, and MUS312–SLX1 and are each required in the absence of DmBLM, presumably because these enzymes cleave spontaneously arising DNA structures that are usually acted upon by DmBLM. Although each of these nucleases has been considered primarily as an enzyme that cuts HJs, it is likely that the toxic intermediates that contribute to lethality also include other structures derived from replication forks. We have suggested models to explain the unique functions for each of these nucleases that become essential when DmBLM is absent. Even if these models are correct, it is likely that they describe only a subset of roles for these enzymes. Further studies of cellular phenotypes that occur in mutants lacking various combinations of these enzymes should provide additional insights into the complexities of replication fork repair and the origins and mechanisms of mitotic recombination.

## Materials and Methods

### Fly Stocks and Culture

Flies were raised on standard cornmeal-based media at 25°C. The following allelic combinations were used: *mus312* mutants were heteroallelic for the null alleles *mus312^D1^* (Q226ter) and *mus312^Z1973^* (K143ter); *mus309* mutants were heteroalleleic for the null alleles *mus309^D2^* (W922ter) and *mus309^N1^* (Δ 2480bp N-terminus) or *mus309^D2^* and the separation-of-function allele *mus309^N2^* (Δ1537 bp N-terminus); *Gen* mutants were hemizygous for *Gen^4325^* (*mus324^Z4325^*) or *Gen^5997^* (*mus324^Z5997^*), over *Df*(*3L*)*Exel6103* (deletes 19 genes in 64C4-64C8, including *Gen*); *mus81* mutants were homozygous (females) or hemizygous (males) for *mus1^Nhe1^*, which has a premature stop codon inserted by targeted recombination [28].

To generate a synthetic deletion of *slx1*, we first made *Df(3R)HKK1* by inducing FLP-mediated recombination between the *FRT* sequences on *P*{*XP*}*d03662*, inserted at 425,462 (coordinates are from chromosome *3L* in release 5.36 of the *Drosophila* genome) and *PBac* [37] *slx1^e01051^*, inserted at 470,260, in the 3′ untranslated region of *slx1* (Figure S2). To complement genes other than *slx1* that were deleted in *Df(3R)HKK1*, we modified the P[acman] clone CH321-44C16 [68], which carries sequences spanning 399,145 to 473,218. We used recombineering to replace 469,261 to 470,077 with a gene conferring bacterial resistance to kanamycin. The deleted region contains almost the entire *slx1* coding sequence, but does not overlap with *MED31*. We were initially unable to get transformants of this large BAC clone, so we also replaced the 39-kb region from 399,284 to 438,520 with the *bla* gene that confers resistance to ampicillin. The remaining insert spans all annotated genes that are deleted in *Df(3R)HKK1*,but is deleted for most of *slx1*. This clone was transformed into a *phiC31 attP* landing site on *3L* (*P*{*CaryP*}*attP2*, in 68A4; injections were done by BestGene, Inc.). We named this integration *Dp(3;3)HKK2*. Finally, we constructed a recombinant chromosome that has *Dp(3;3)HKK2* and *Df(3R)HKK1*. This chromosome is therefore euploid except for the loss of *slx1*. Flies homozygous for this chromosome are viable and fertile.

### Apoptosis in Imaginal Discs

Discs were harvested in Ringer's buffer from wandering third instar larvae and fixed in 4% formaldehyde in PBST (0.1% Triton-X in PBS) for 45 min. After washing in PBST, the discs were blocked in 5% bovine serum albumin in PBST 1 hr at room temp. They were then stained overnight at 4°C with 1∶500 anti-Cleaved Caspase-3 (Cell Signaling #966S) in PBST. The following day, the discs were stained two hours at room temperature with 1∶1000 the 2° Alexa Fluor 488 goat anti-rabbit (Molecular Probes #A11034). Discs were then washed, fine-dissected, and mounted on a slide with Fluoromount-G (SouthernBiotech #0100-01) and sealed with nail polish. Images were taken with a Nikon Eclipse E800 fluorescent microscope.

### Larval Neuroblast Squashes

Third instar larvae brains were dissected and soaked in 0.1 mM colchicine in 0.7% saline for 1.5 hrs, followed by 8 min in 0.5% sodium citrate. Brains were fixed for 20 sec in a 5.5∶5.5∶1 solution of acetic acid: methanol: water. Brains were transferred to a slide and treated with 45% acetic acid for 2 min, then squashed under a siliconized coverslip. The coverslip/slide was placed on dry ice for 10 min, then the coverslip was flicked off and the slide washed in −20°C ethanol then dried for storage at 4°C. The slide was rehydrated for 5 min in 2x SSC (300 mM NaCl, 30 mM sodium citrate, pH 7.0), then stained in 2x SSC plus 1∶10,000 DAPI (1mg/mL) for 5 min, then washed in 2xSSC and air-dried. The slide was mounted with Fluoromount-G (SouthernBiotech #0100-01) and sealed with nail polish. Images were taken with a Nikon Eclipse E800 fluorescent microscope.

### Salivary Gland Analysis

Salivary glands were dissected from third instar larvae in Ringer's buffer and fixed for 45 min in 4% formaldehyde in PBST (0.1% Triton-X in PBS). After PBST washes, the glands were stained with 1∶1000 DAPI (1mg/ML) 5 min and washed again. Glands were mounted on slides using Fluoromount-G (SouthernBiotech #0100-01), sealed with nail polish, and imaged on a Nikon Eclipse E800 fluorescent microscope.

## Supporting Information

Figure S1 Double-strand break repair models. In this figure, yellow lines are the broken chromatid and black lines are the repair template. Arrowheads point toward 3′ ends. The double-strand break (DSB; i) is first resected to generate 3′ single-stranded overhangs (ii). One of these invades a repair template and primes repair synthesis (iii). In mitotically proliferation cells, this structure is typically dissociated, making the newly synthesized sequence available to anneal to the other resected end; this generates a non-crossover (NCO) product (iv). This mechanism is refered to as synthesis-dependent strand annealing (SDSA). In some cases, the strand displaced from the template by synthesis can anneal to the other resected end, which can then prime additional repair synthesis. Ligation of the free ends produces a double-Holliday junction (dHJ) structure (vi). The dHJ can undergo dissolution (convergent branch migration and decatenation) to generate an NCO (vii). Alternatively, the dHJ can undergo resolution. Since each HJ can be cut in one of two orientations, there are four possible outcomes. Two of these are shown. Cutting different strands at each HJ (viii, arrowheads indicate nicks) generates a crossover (CO; ix), but cutting the same strands (x) generates an NCO (xi). Products are drawn prior to mismatch repair and final ligation.
(TIF)

Figure S2 Mutations in *slx1*. (A) Genomic region 400,000 to 473,000 on *3R* is shown. The two transposable element insertions used to generate *Df(3L)HKK1* are indicated above the scale bar. The extent of this deletion (red line) and of *Dp(3;3)HKK2* (purple line) are indicated. The genespans of *slx1* (blue), *MED31* (green), and other annotated genes (gray) are shown below. (B) Zoom of the region spanning *slx1* and *MED31*. This diagram shows the overlap between the non-coding exons of *MED31* and *slx1* (the first four residues of SLX1 are encoded on the second exon, which overlaps the first *MED31* exon). Additional *MED31* transcripts are also annotated, but not shown here. The region of *slx1* that is deleted in *Dp(3;3)HKK2* is indicated with a dashed, purple line. (C) An alignment of the GIY-YIG nuclease domain from SLX1 of *Drosophila melanogaster* (residues 23-106), *Homo sapiens* (13-94), *Saccharomyces cerevisiae* (13-94), and *Arabidopsis thaliana* (27-107) is presented. The position of the F92I missense mutation is indicated (red arrow).
(TIF)
