# Supplementary figures and images for: Three Structure-Selective Endonucleases Are Essential in the Absence of BLM Helicase in Drosophila
Source: PLoS Genet. 2011 Oct 13;7(10):e1002315. doi: 10.1371/journal.pgen.1002315 (PMC3192830; doi:10.1371/journal.pgen.1002315)

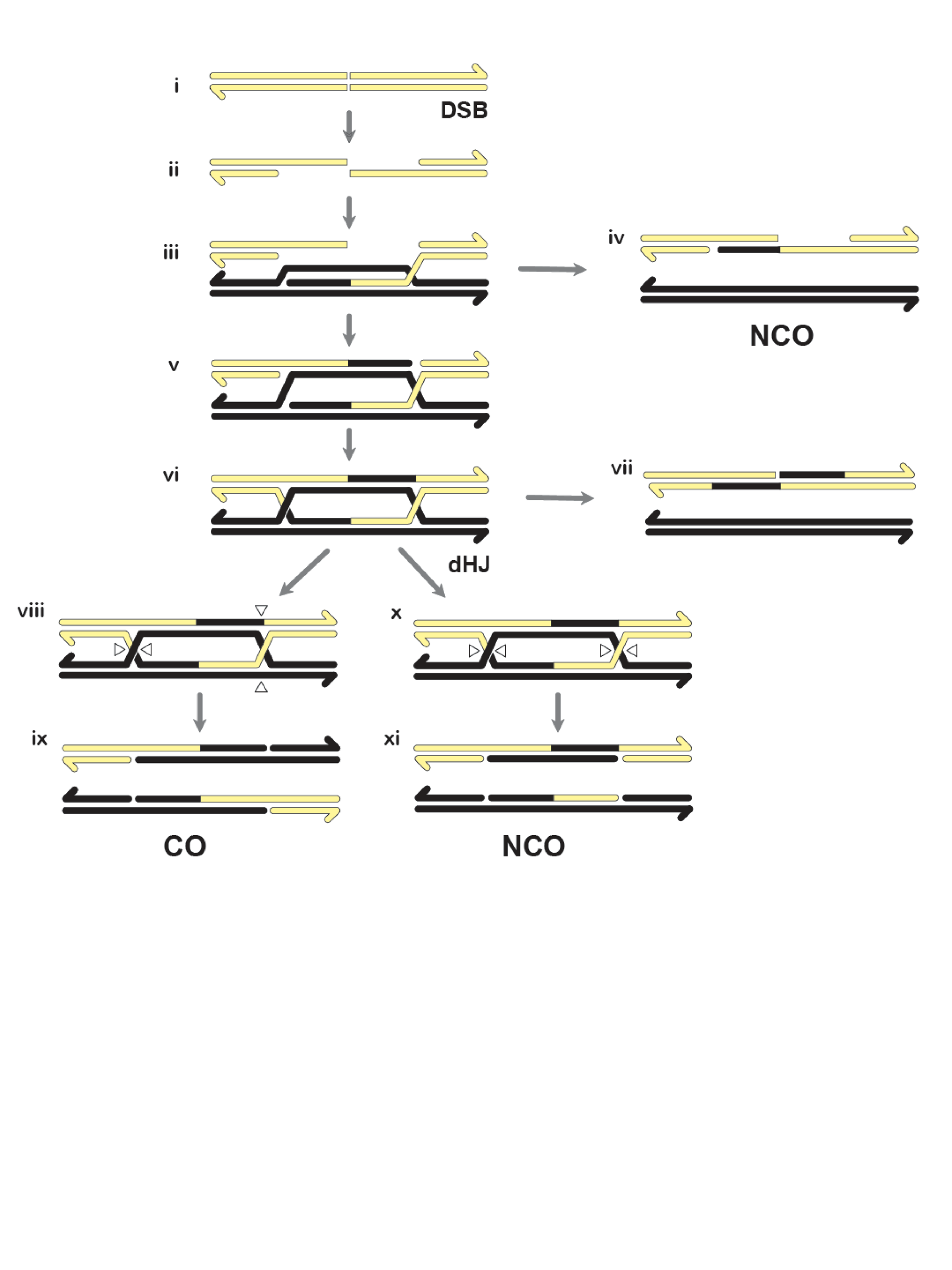

Supplement: Figure S1 — Double-strand break repair models. In this figure, yellow lines are the broken chromatid and black lines are the repair template. Arrowheads point toward 3′ ends. The double-strand break (DSB; i) is first resected to generate 3′ single-stranded overhangs (ii). One of these invades a repair template and primes repair synthesis (iii). In mitotically proliferation cells, this structure is typically dissociated, making the newly synthesized sequence available to anneal to the other resected end; this generates a non-crossover (NCO) product (iv). This mechanism is refered to as synthesis-dependent strand annealing (SDSA). In some cases, the strand displaced from the template by synthesis can anneal to the other resected end, which can then prime additional repair synthesis. Ligation of the free ends produces a double-Holliday junction (dHJ) structure (vi). The dHJ can undergo dissolution (convergent branch migration and decatenation) to generate an NCO (vii). Alternatively, the dHJ can undergo resolution. Since each HJ can be cut in one of two orientations, there are four possible outcomes. Two of these are shown. Cutting different strands at each HJ (viii, arrowheads indicate nicks) generates a crossover (CO; ix), but cutting the same strands (x) generates an NCO (xi). Products are drawn prior to mismatch repair and final ligation. (TIF) [file pgen.1002315.s001.tif]

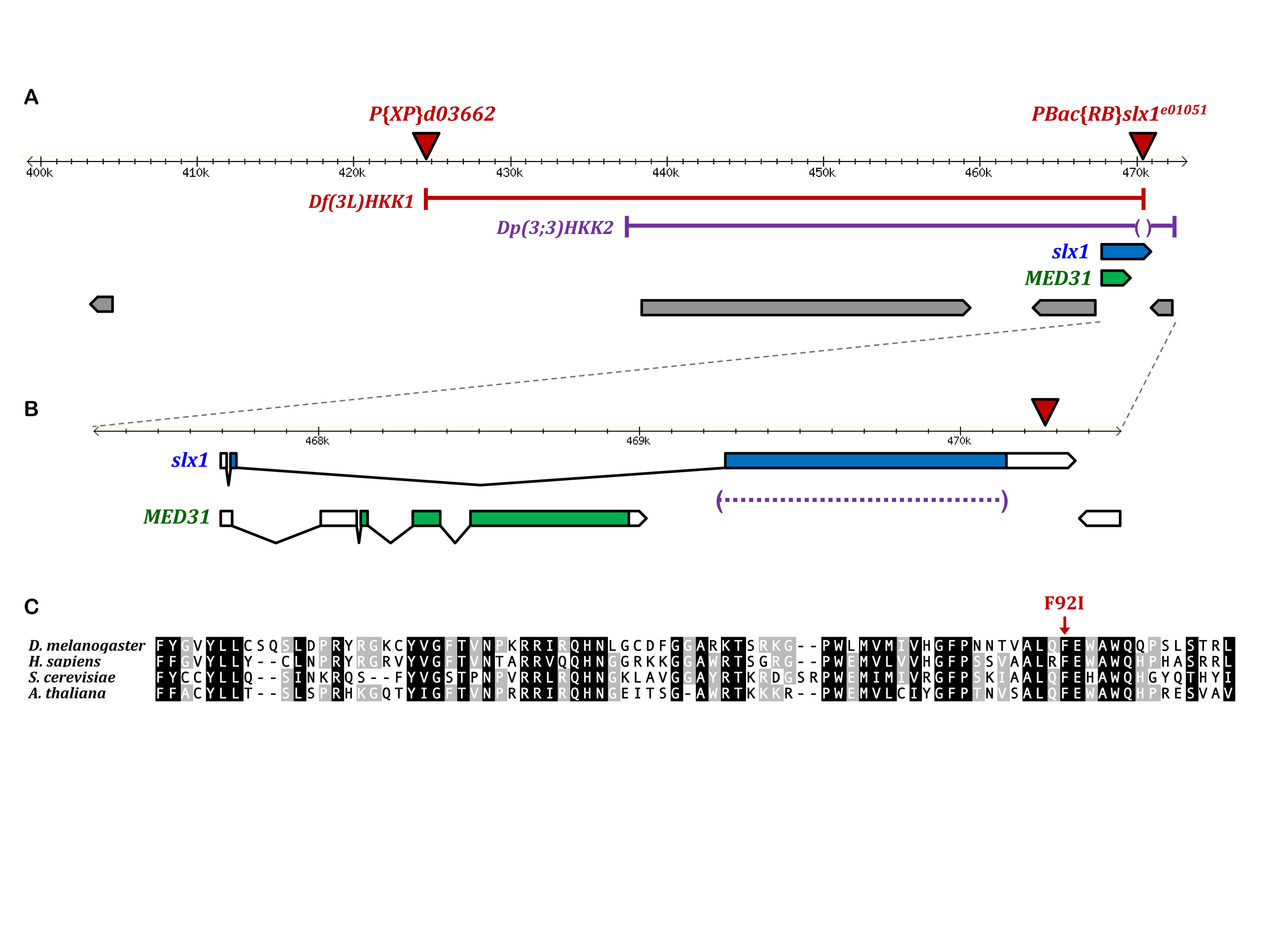

Supplement: Figure S2 — Mutations in slx1. (A) Genomic region 400,000 to 473,000 on 3R is shown. The two transposable element insertions used to generate Df(3L)HKK1 are indicated above the scale bar. The extent of this deletion (red line) and of Dp(3;3)HKK2 (purple line) are indicated. The genespans of slx1 (blue), MED31 (green), and other annotated genes (gray) are shown below. (B) Zoom of the region spanning slx1 and MED31. This diagram shows the overlap between the non-coding exons of MED31 and slx1 (the first four residues of SLX1 are encoded on the second exon, which overlaps the first MED31 exon). Additional MED31 transcripts are also annotated, but not shown here. The region of slx1 that is deleted in Dp(3;3)HKK2 is indicated with a dashed, purple line. (C) An alignment of the GIY-YIG nuclease domain from SLX1 of Drosophila melanogaster (residues 23-106), Homo sapiens (13-94), Saccharomyces cerevisiae (13-94), and Arabidopsis thaliana (27-107) is presented. The position of the F92I missense mutation is indicated (red arrow). (TIF) [file pgen.1002315.s002.tif]
